# Next Generation Device Grade Silicon-Germanium on Insulator

**DOI:** 10.1038/srep08288

**Published:** 2015-02-06

**Authors:** Callum G. Littlejohns, Milos Nedeljkovic, Christopher F. Mallinson, John F. Watts, Goran Z. Mashanovich, Graham T. Reed, Frederic Y. Gardes

**Affiliations:** 1 Optoelectronics Research Centre, University of Southampton, Southampton, SO17 1BJ, UK; 2 The Surface Analysis Laboratory, Department of Mechanical Engineering Sciences, University of Surrey, Guildford, Surrey, GU2 7XH, UK

## Abstract

High quality single crystal silicon-germanium-on-insulator has the potential to facilitate the next generation of photonic and electronic devices. Using a rapid melt growth technique we engineer tailored single crystal silicon-germanium-on-insulator structures with near constant composition over large areas. The proposed structures avoid the problem of laterally graded SiGe compositions, caused by preferential Si rich solid formation, encountered in straight SiGe wires by providing radiating elements distributed along the structures. This method enables the fabrication of multiple single crystal silicon-germanium-on-insulator layers of different compositions, on the same Si wafer, using only a single deposition process and a single anneal process, simply by modifying the structural design and/or the anneal temperature. This facilitates a host of device designs, within a relatively simple growth environment, as compared to the complexities of other methods, and also offers flexibility in device designs within that growth environment.

Si_1-x_Ge_x_ has many attractive characteristics which can be exploited for numerous applications including wavelength sensitive photonic devices^1^, high mobility complementary metal oxide semiconductor (CMOS) devices^2^ and lattice matching for epitaxial III-V growth^3^,^4^. It possesses full miscibility across its entire composition range which allows for the tuning of properties such as the bandgap and lattice constant between those of bulk Si and bulk Ge. This means that depending on the composition, SiGe alloys can be either optically absorbing or transparent at telecommunication wavelengths (1550 nm or 1310 nm) which enables the fabrication of active devices for both modulation^5^,^6^ and detection^6^,^7^. SiGe has both a higher hole and electron mobility than Si, meaning that it will ultimately lead to faster electronic devices, e.g. transistors^8^, and is also a CMOS compatible material.

A number of methods have been proposed to produce silicon-germanium-on-insulator (SGOI) films on Si wafers, including layer transfer^9^,^10^ and Ge condensation^10^,^11^. A complementary technique for fabricating localized SGOI regions on a Si wafer is rapid melt growth (RMG). RMG, also referred to as liquid phase epitaxy (LPE), is a technique that was invented in the 1960's^12^ and further developed in the 1970s for the fabrication of detectors^13^, LEDs^14^ and laser diodes^15^. The technique, originally used for III–V crystal growth, was pioneered by Liu *et al.*^16^ for localized germanium-on-insulator (GOI) growth in 2004 and has since been studied by various groups^17^,^18^ and adapted for SGOI growth^19^,^20^. In order to realise RMG a polycrystalline Ge layer is deposited onto a patterned insulating layer (in this case SiO_2_), with areas of the Si substrate exposed to act as a seed for crystal regrowth. The Ge is patterned and encapsulated in micro-crucibles so that when it is melted and subsequently cooled it mimics the template of the Si crystal structure. This process is described in Figure 1. To date, RMG of Ge has been demonstrated for gate all around P-MOSFETs^21^,^22^, P-channel finFETs^23^ and waveguide integrated heterojunction photodiodes^24^. These devices demonstrate the possibility of using RMG to obtain high quality Ge single crystal layers on localised insulator islands located on top of silicon substrates or silicon-on-insulator (SOI) substrates. This enables a bridge between electronic components and photonic components; the latter group IV based components being predominantly confined to the SOI platform^25^,^26^,^27^,^28^,^29^,^30^,^31^. This vision is clearly demonstrated by Going *et al.* in a gate photoMOSFET^32^ where a Ge gated NMOS phototransistor is integrated on a SOI photonics platform.

RMG is very attractive for the heterogeneous integration of SiGe based devices on insulator for electronics and photonics applications because it is possible to grow defect-free single crystal material, by the mechanism shown in Figure 2a, with the condition that the regrowth propagation speed is sufficient enough to avoid random nucleation in the liquid ahead of the epitaxial growth front^20^. This high material quality can lead to significant improvements in device characteristics such as leakage current and quantum efficiency. However, until now accurate control of the SiGe composition in the RMG layers has proved to be extremely challenging. This is due to the large separation between the SiGe composition in the solid and liquid phase at any given temperature, shown by the SiGe phase diagram in Figure 2b. This large composition separation between phases results in preferential Si rich solid formation at the growth front with rejection of Ge into the liquid, leading to a gradation of the SiGe composition in the regrowth direction of a straight strip as the Si is consumed^19^,^20^. By using the tailored tree-like structure shown in Figure 2c we have demonstrated for the first time (Figure 3a) that the SiGe composition (shown on the graph as *I(SiGe)/I(GeGe)*) can be engineered to a near constant value along the central strip of the tree-like structure, with the graded composition characteristics previously demonstrated in straight strips appearing only in the branches of the tree. This is to be expected since the branches of the tree-like structure are effectively straight strips seeding from the central part of the tree-like structure. In practice the branches would be removed once the RMG process has been performed, leaving only the constant composition SGOI material for device fabrication. Throughout this paper the centre strip of the tree-like structure will be referred to as ‘central strip' and a straight strip, with no branches, will be referred to as ‘straight strip'.

## Results

The SiGe composition in our structures was characterized using 532 nm Raman spectroscopy, as well Auger Electron Spectroscopy (AES) in order to confirm the integrity of the data. The Ge concentration can be calculated from the Raman spectra using the equation proposed by Mooney *et al.*^33^ by taking the ratio of the Si-Ge mode intensity and Ge-Ge mode intensity (the peaks located at approximately 380 cm^−1^ and 300 cm^−1^ respectively) as described by Equation (1):
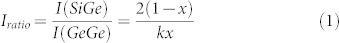
Here, *I(SiGe)* and *I(GeGe)* are the integrated intensities of the Si-Ge and Ge-Ge modes respectively, *x* is the Ge concentration and *k*, which is dependent on the excitation wavelength, is an experimental setup specific constant that can be determined from samples of known composition. For the purpose of this paper only the ratios of the integrated intensities, *I_ratio_*, are considered. Nevertheless, for high Ge compositions (*x* > 0.75) the correlation between *I_ratio_* and *x* is almost linear for all values of *k* quoted in the literature rendering this analysis an accurate representation of the actual SiGe composition^17^. This has been confirmed by comparing secondary ion mass spectrometry (SIMS) composition data to *I_ratio_* for some blanket SiGe on Si samples, grown using reduced pressure chemical vapour deposition (RPCVD), with compositions in the range 0.75 < *x* < 1. A similar characterization process was carried out using AES. For this analysis the peak ratios between Si and Ge (found at approximately 1615 eV and 1145 eV respectively) were calculated after a linear background subtraction was performed. Again it is expected that this ratio closely relates to the SiGe composition^34^.

In Figure 3b both the Raman *I_ratio_* and the AES *Si/Ge Peak ratio* are shown as a function of distance from the seed for the centre strip of the tailored structure and a straight strip, with the tree branches omitted, of the same width. This clearly demonstrates a dramatic improvement of the composition consistency when using the tailored structure. This figure establishes that the AES data is in agreement with the Raman data, within measurment tolerances, so we have displayed only the Raman data for all other samples. For quantification of Si the more intense Si LMM Auger transition is usually used. This would provide a more precise value for concentration as its peak height is more sensitive to concentration. However this transition cannot be used in this system as a consequence of the large energy overlap between the Ge MNN and Si LMM transitions. As a result the lower sensitivity Si KLL transition must be used instead. However, at low Si concentrations this peak is very weak and the region must be force fitted to provide a value of intensity. Therefore the discrepancy between the AES results and the Raman results at low Si concentrations is likely due to the poor sensitivity of the Si KLL peak to concentration changes in this composition range.

It is also important to consider the penetration depths associated with the respective measurement techniques. AES has a sampling depth of several nanometers and thus will show only surface information. A 532 nm laser has a sampling depth of several tens of nanometers in high Ge percentage SiGe but is dependent on the composition^35^. Therefore it is possible that there is a vertical distribution of composition but this is predicted to be negligible since the epitaxial growth front propagates laterally and not vertically.

Figure 3c shows that we have also demonstrated that the composition of the localized SGOI structures can be controlled by the peak anneal temperature, with more Si rich alloys formed at higher anneal temperatures.

The maximum strip width for which high quality SGOI was achieved was 5 μm. At greater widths the surface tension between the SiGe melt and the surrounding insulating layer resulted in agglomeration of the SiGe.

Threading dislocations, caused by the lattice constant mismatch between Si and Ge, are clearly observed in the transmission electron microscope (TEM) images shown in Figure 4a, but are confined to the seed area and do not propagate along the SiGe strip. The high resolution cross-section confirms single crystal, defect free SGOI along the centre strip of the tree structure. Ge diffusion into the Si substrate can also be observed. Electron back-scatter diffraction (EBSD) measurements, shown in Figure 4b, confirm the single crystal orientation of the SiGe tailored structures.

## Discussion

In order to discuss the cause of these results it is firstly important to understand the recrystallization and Si diffusion mechanisms that are exhibited in the conventional RMG process. Once the Ge melts upon heating to become liquid, Si diffusion into the Ge from the seed dramatically increases to form a liquid SiGe pool. It can be approximated that the SiGe composition is uniform in the liquid pool due to the high diffusivities of both Si^36^ and Ge^37^ in liquid SiGe. At a given alloy composition, determined by the anneal temperature, the SiGe will begin to solidify in the seed area, due to the slightly higher Si concentration found here, and mimic the crystal structure of the underlying Si substrate. Since the diffusivity of both Si and Ge in solid SiGe is many orders of magnitude lower than in liquid SiGe it can be approximated that there is no diffusion in the solid phase. Therefore, once the SiGe in the seed area has solidified the Si supply from the substrate is cut off leading to a finite Si ‘pool' available in the SiGe melt. As described above, the SiGe phase diagram shows that there is a preferential growth of Si rich solid which results in complete depletion of the Si ‘pool' before the end of a straight strip. However if radiating elements are added to the strip to form a tailored tree-like structure (see Figure 2c) the cooling rate of the structure is increased and therefore the regrowth front propagation velocity is increased. This means that complete diffusion of the rejected Ge into the bulk of the liquid does not occur and therefore the SiGe composition becomes more consistent.

At higher temperatures more Si diffusion into the Ge layers is apparent and since recrystallization will not commence until the Si concentration reaches the solidification point determined by the SiGe phase diagram, a more Si rich composition is achieved (see Figure 3c). This would therefore enable tuning of the band edge and lattice parameters of the layer to suit the design requirements simply by modifying the anneal temperature. It is to be noted here that, whilst the quoted temperatures are accurate relative to one another, they should be considered arbitrary temperatures in the absolute sense, because they were recorded using a pyrometer measuring the infra-red radiation from the back of the Si substrate during annealing. This recorded temperature does not match the SiGe temperature since the optical absorption coefficients of the two materials are different and will vary depending on the alloy composition.

The fact that the SiGe composition is consistent in the centre strip of the tree-like structure but then follows the characteristics of a straight strip in the branches suggests that the regrowth front propagates firstly along the centre stripand secondly along the individual branches, with the centre strip acting as a seed for each branch. In order to confirm this hypothesis an experiment was designed to study the solidification of the tailored structures. In this experiment we used a poly-silicon (poly-Si) seed on a thick insulating layer rather than a bulk Si seed. This has the effect of slowing down the cooling rate, and therefore the regrowth front propagation velocity, of the SiGe structures, whilst still maintaining single crystal regrowth^17^, by reducing the heat sinking properties of the bulk Si wafer. The result is that random nucleation occurs in the liquid SiGe ahead of the regrowth front. The nucleation points are observable on scanning electron microscope (SEM) images, as shown in Figure 5. The low nucleation count observed in the centre strip of the tree-like structure compared with the high nucleation count observed in the branches is consistent with our hypothesis that the centre strip solidifies prior to the branches, because only in the branches is the regrowth rate slow enough to allow random nucleation to occur.

In conclusion, the simple method described here for fabrication of localized SGOI structures could act as the blueprint for the coexistence of next generation SiGe electronic and photonic devices on the same wafer. The SiGe composition engineering that produces single crystal, defect free, SiGe layers could enable the fabrication of a multitude of devices requiring different compositions of SiGe on the same wafer in a single deposition step and a single anneal step. Thanks to this methodology the composition of SiGe can now be varied according to the anneal temperature and/or the material structure enabling alloy composition engineering dictated by structural design and not by the deposition or growth mechanism. This method leads to a simplified (single deposition), design enabled fabrication process for the integration of a plethora of electronic and CMOS compatible photonic devices on a standard Si substrate providing a path for the seamless integration of electronics and photonics at a low cost.

## Methods

### Rapid melt growth fabrication process

**Figure 1:**
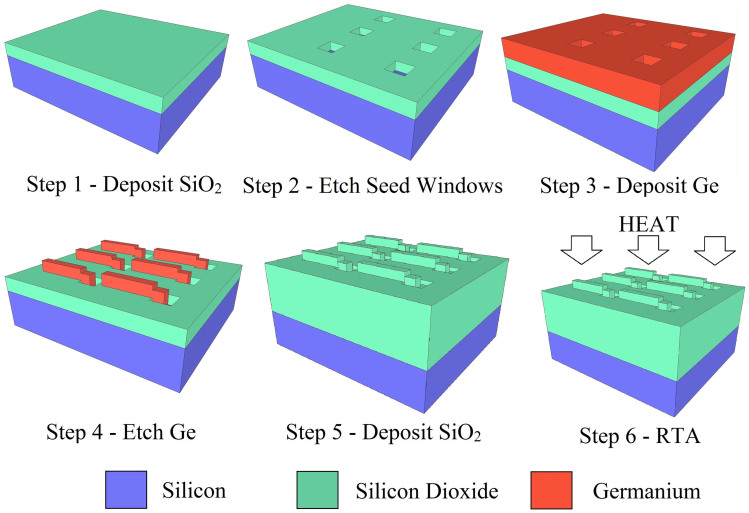
Summary of the SiGe rapid melt growth fabrication process.

**Figure 2:**
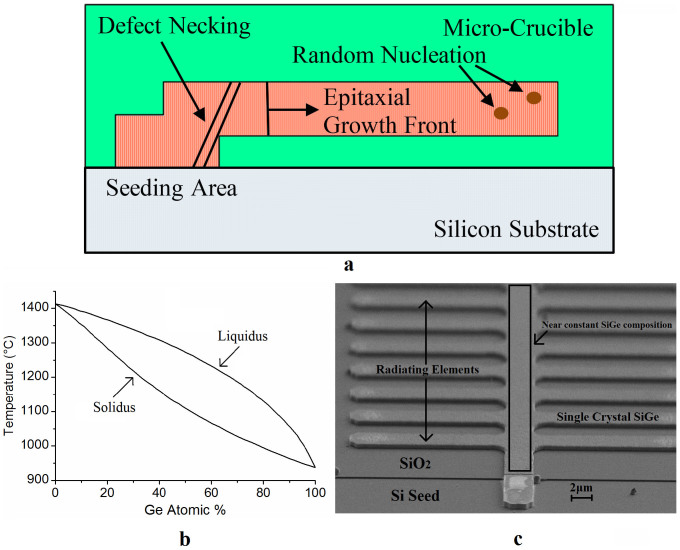
Summary of the SiGe recrystallization process. (a), A cross-section schematic of the recrystallization process showing the growth front initiating at the Si seed, which is essential to ensure single crystal epitaxial regrowth, and propagating along the SiGe structure. Growth is initiated at the seed for two reasons: firstly the Si substrate acts as a heat sink ensuring more rapid cooling in the seed area; secondly, Si diffusion into the Ge structure increases the solidification temperature so that material with a slightly higher Si composition solidifies first (i.e. areas nearer the Si seed). Random nucleation occurs when the growth front propagation speed is too slow. (b), Phase diagram of SiGe alloys showing separation of the solidus and liquidus curves – adapted from ref. 38. (c), An SEM image of the tailored tree-like structure showing the radiating elements which result in a near constant SiGe composition in the central strip.

**Figure 3:**
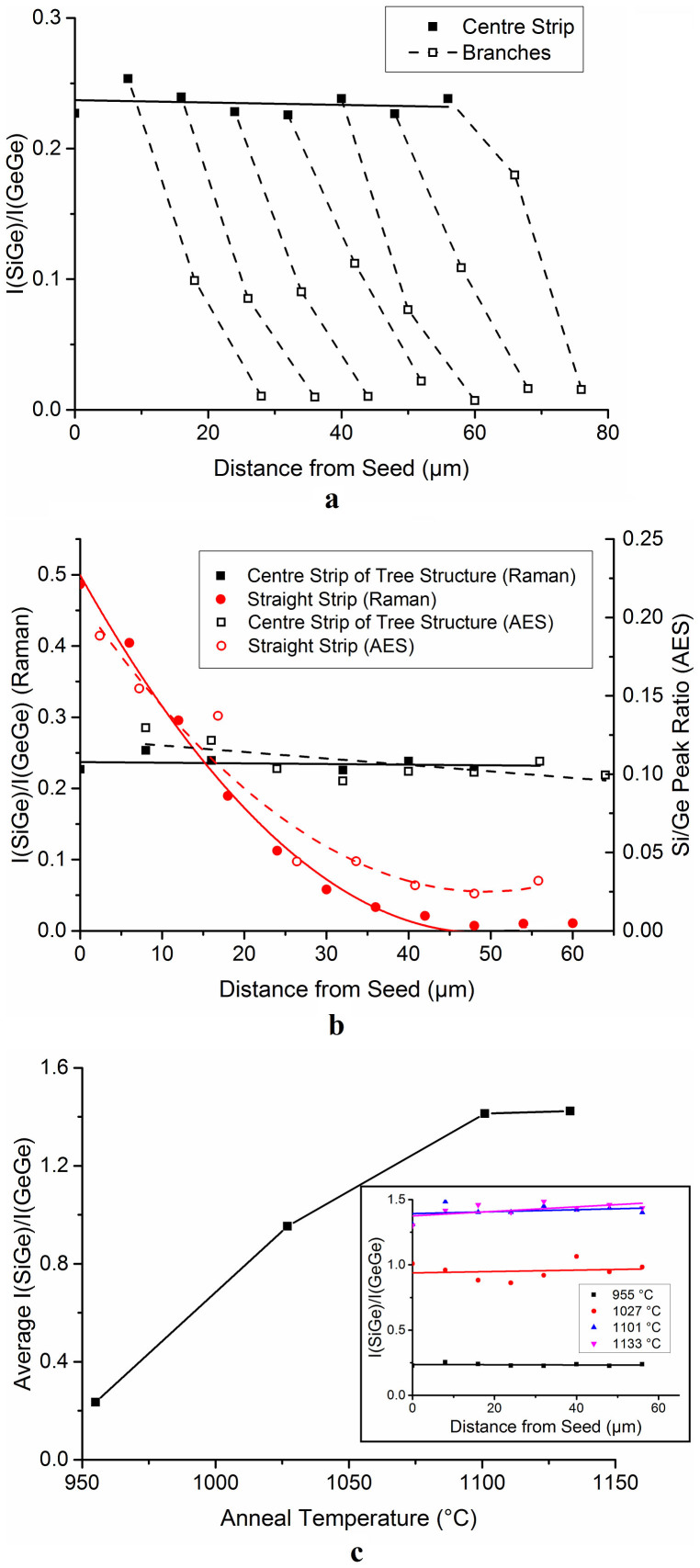
Study of the SiGe composition in tailored tree-like structures. (a), Raman *I_ratio_* as a function of distance from the Si seed for a tailored tree-like structure annealed at 955°C, showing near constant SiGe composition in the central strip (solid), and graded composition characteristics in the branches (dashed). Each dashed line represents an individual branch. (b), A comparison of the Raman *I_ratio_* on the left hand axis (solid) and AES *Si:Ge peak ratio* on the right hand axis (dashed) for the central strip of a tailored tree-like structure (black squares) and a straight strip, with the branches omitted (red circles), as a function of distance from the Si seed. This shows the dramatic improvement in composition consistency when using the tailored tree-like structure. The straight strip data has been standardized using data from 10 μm, 20 μm, 50 μm and 100 μm long strips in order to match the length of the 60 μm long tailored tree-like structure; as described in ref. 19. All structures were annealed at 955°C. (c), Average Raman *I_ratio_* along the centre strip of a tailored tree-like structure as a function of anneal temperature showing increased Si composition at higher temperatures due to the increased Si diffusion from the seed. Inset is a plot of the Raman *I_ratio_* as a function of distance from the seed for a range of anneal temperatures from which the average *I_ratio_* values were calculated.

**Figure 4:**
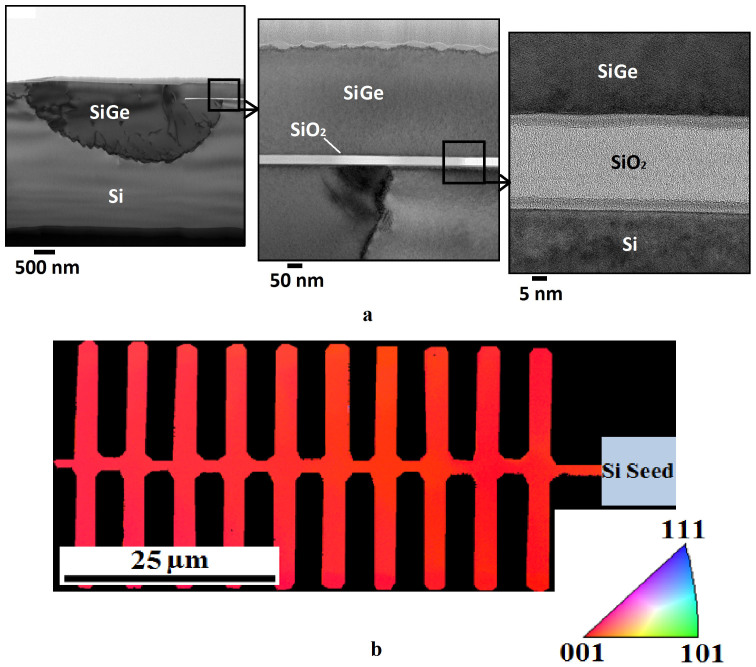
SGOI crystal quality analysis. (a), cross-section TEM images of RMG SiGe annealed at 955°C showing threading dislocations confined to the seed area and defect free single crystal SGOI away from the seed area. The layer on top of the SiGe is e-beam platinum which has been added for protection during FIB milling. (b), EBSD scan of the tailored tree-like structure showing complete (001) SiGe crystal orientation, matching that of the Si substrate.

**Figure 5:**
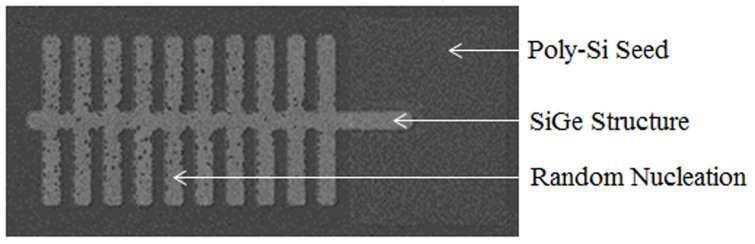
SEM image of RMG SiGe with poly-Si seed. The poly-Si seed deposited on a thick (2 μm) SiO_2_ layer does not have the heat sinking capabilities of a bulk Si seed resulting in a slower regrowth front propagation velocity in the SiGe structure; therefore enabling random nucleation to occur in the SiGe. This image suggests that the central strip of the structure recrystallizes first, due to the low nucleation count, and only then the branches recrystallize, due to the high nucleation count in the branches.

The SiGe tailored structures were grown on 6 inch (100) Si wafers. The wafers were cleaned using a conventional RCA clean prior to processing to remove any contaminants from the substrate surface. A 50 nm SiO_2_ layer was then deposited using plasma enhanced chemical vapour deposition (PE-CVD). The SiO_2_ layer was then patterned using standard UV photolithography and a dilute (20:1) HF wet etch in order to expose the underlying Si to act as a seed for the SiGe recrystallization process. A 400 nm Ge layer was deposited using a non-selective PE-CVD process and patterned using standard UV photolithography and an inductively coupled plasma (ICP) etch, leaving Ge structures overlapping the Si seeds. A 1 μm SiO_2_ layer was then deposited by PE-CVD in order to encapsulate the Ge structures, forming micro-crucibles. The wafers were subsequently heated in a rapid thermal annealer (RTA) in order to melt the encapsulated Ge and initiate recrystallization. The temperature of the RTA was stabilized at 500°C before ramping to the maximum temperature (in the range 955°C to 1133°C) at a rate of approximately 100°C/s. The wafers were held at the maximum temperature for 1 second before cooling naturally. Finally, the top SiO_2_ layer was removed using a 20:1 HF wet etch for material characterization. This process is summarized in Figure 1.

### Material characterization

The SiGe composition has been characterized firstly using 532 nm Raman spectroscopy with a spot size of approximately 0.5 μm. The ratio of the Si-Ge and Ge-Ge mode intensities, *I_ratio_*, for each point has been calculated by fitting a Lorentzian curve to each peak to calculate the area under the peak. In order to confirm the integrity of this data Auger electron spectroscopy (AES) was also performed using a Thermo Scientific Microlab 350 Scanning Auger Microscope, with a spot size of 20 nm. High resolution Si *KLL* and Ge *LMM* spectra were collected from 1300–1650 and 650–1205 eV, respectively, with a step size of 2 eV, a retard ratio of 4 and a dwell time of 100 ms per channel. The air formed surface oxide and surface carbon contamination were removed with 20 seconds of argon ion sputtering using a 3 kV beam energy and 1 μA sample current prior to measurement. A linear background subtraction is performed over the peak ranges 1300–1650 and 650–1205 eV for silicon and germanium respectively, which is necessary as the large background distorts the true peak intensity. Cross section TEM lamellae were prepared by EAG Labs using an in-situ focused ion beam (FIB) lift out technique. For protection the samples were coated with local e-beam and ion-beam platinum prior to FIB milling. EBSD measurements were also performed by EAG Labs. The sample surface was etched with a gallium ion beam prior to EBSD imaging. The SEM images were collected using a Zeiss NVision 40 FIB System.

## Author Contributions

C.G.L. and F.Y.G. conceived and designed the experiments. C.G.L. performed the fabrication and Raman characterization. C.G.L., F.Y.G. and M.N. carried out the data analysis of the Raman spectra. C.F.M. performed the AES measurements. C.F.M, J.F.W, F.Y.G and C.G.L carried out the AES data analysis. F.Y.G performed the SEM imaging. All authors contributed to the manuscript preparation. G.Z.M. and G.T.R. supervised the project.
